# Molecular detection, genetic characterization, and phylogenetic relationships of <em>Theileria orientalis</em> in cattle and associated ticks from Wasit Province, Iraq

**DOI:** 10.14202/vetworld.2026.2850-2863

**Published:** 2026-07-10

**Authors:** Abbas H. K. Sray, Ghassan J. K. Al-Abedi, Thuraya Khaled Abdulwahed, Israa M. Essa, Zeid Alsadoon, Hasanain A. J. Gharban

**Affiliations:** 1 Department of Parasitology, College of Veterinary Medicine, University of Wasit, Wasit 52001, Iraq.; 2 Medical Laboratories Techniques Department, College of Health and Medical Techniques, University of Kut, Wasit 52001, Iraq.; 3 Department of Medical Physics, College of Science, University of Kut, Wasit 52001, Iraq.; 4 Department of Public Health, College of Veterinary Medicine, University of Basrah, Basra 61001, Iraq.; 5 Department of Parasitology, College of Veterinary Medicine, University of Wasit, Wasit, Iraq.; 6 Department of Internal and Preventive Veterinary Medicine, College of Veterinary Medicine, University of Wasit, Wasit 52001, Iraq.

**Keywords:** 18S rRNA, cattle, genetic characterization, Iraq, molecular epidemiology, phylogenetic analysis, Theileria orientalis, tick-borne disease

## Abstract

**Background and Aim:**

*Theileria orientalis* is an emerging tick-borne hemoprotozoan that has become a major cause of bovine theileriosis, leading to anemia, reduced productivity, and substantial economic losses in cattle. Despite increasing reports from neighboring countries, molecular epidemiological information and phylogenetic evidence for *T. orientalis* in Iraq remain scarce. This study aimed to determine the molecular prevalence of *T. orientalis* in cattle and their naturally infesting ticks in Wasit Province, Iraq; identify host-associated risk factors; and characterize the genetic diversity and phylogenetic relationships of local isolates relative to global reference strains.

**Materials and Methods:**

A cross-sectional study was conducted on 170 naturally tick-infested cattle from Wasit Province, Iraq, during July-August 2025. Whole blood and corresponding tick samples were collected from each animal. Genomic DNA was extracted and screened for *T. orientalis* using conventional polymerase chain reaction targeting the *18S rRNA* gene. Positive amplicons were sequenced by Sanger sequencing, followed by sequence alignment, GenBank submission, and phylogenetic analysis using MEGA 11. Associations between infection and animal-related risk factors were evaluated using Chi-square analysis, odds ratio, relative risk, and 95% confidence intervals.

**Results:**

Molecular analysis detected *T. orientalis* DNA in 21/170 (12.35%) cattle and 16/170 (9.41%) tick samples. Infection prevalence differed significantly according to age and sex. Cattle aged >3-6 years exhibited the highest infection risk, whereas male cattle showed significantly greater positivity and relative risk than females. Sequence analysis demonstrated high genetic conservation among Iraqi isolates. Phylogenetic reconstruction revealed that all cattle isolates clustered closely with a Turkish *T. orientalis* isolate (HQ197736.1), whereas tick isolates showed close genetic relationships with Turkish isolates (HQ197736.1 and OR211412.1) and a Chinese isolate (PQ207062.1), suggesting regional genetic connectivity and possible transboundary circulation of *T. orientalis* lineages.

**Conclusion:**

This study provides the first molecular detection and comprehensive phylogenetic characterization of *T. orientalis* simultaneously in cattle and associated tick vectors from Wasit Province, Iraq. The findings establish the first molecular evidence linking genetically related cattle and tick isolates in the country and provide an important baseline for understanding the epidemiology of oriental theileriosis. Expanded nationwide molecular surveillance, genotype-specific characterization using additional genetic markers, and integrated tick management programs are warranted to improve disease monitoring and reduce the impact of *T. orientalis* infection on the Iraqi cattle industry.

## INTRODUCTION

Blood parasites of cattle are microscopic pathogens transmitted primarily by ticks and cause substantial morbidity, mortality, and economic losses through reduced productivity, impaired animal health, and, in some cases, the emergence of drug-resistant strains [1, 2]. Among these pathogens, bovine theileriosis is one of the most important tick-borne diseases and is caused by several *Theileria* species, each exhibiting distinct host specificity, geographic distribution, and pathogenicity [3]. For example, *Theileria annulata* infects cattle and other susceptible hosts across tropical, subtropical, and Mediterranean regions, causing severe tropical theileriosis with high morbidity and mortality, whereas *T. parva* is predominantly distributed in sub-Saharan Africa and is responsible for the highly fatal East Coast fever in cattle [4, 5].

*T. orientalis* has a worldwide distribution and primarily infects cattle, typically causing a mild, non-transforming, and frequently asymptomatic infection. Nevertheless, the parasite has emerged as an important cause of bovine theileriosis because it can induce hemolytic anemia, reduced milk production, decreased weight gain, reproductive losses, and considerable economic damage to the cattle industry [6, 7]. The genus *Theileria* possesses a complex life cycle involving schizonts in lymphoid cells and piroplasms within erythrocytes of vertebrate hosts, whereas ticks become infected by ingesting parasitized erythrocytes during blood feeding [8, 9]. Within the tick gut, erythrocytes are lysed, releasing piroplasms that differentiate into gametes and subsequently fuse to form zygotes. These zygotes penetrate the gut epithelium, develop into kinetes, migrate to the salivary glands, and undergo sporogony to produce infective sporozoites. During subsequent blood feeding, sporozoites are transmitted through tick saliva into the mammalian host, where they invade leukocytes, develop into schizonts, and eventually produce erythrocytic piroplasms [10–12]. Although acute clinical signs such as pyrexia and anemia may be absent or transient, *T. orientalis* infections often persist throughout the host's lifetime, with recrudescence occurring during periods of physiological or environmental stress, including lactation, pregnancy, and sudden environmental changes [13, 14].

Accurate diagnosis of *T. orientalis* infection is essential for effective epidemiological surveillance and disease control. Conventional microscopic examination of Giemsa-stained blood smears has limited sensitivity and specificity, particularly in animals with low parasitemia or chronic infections. Consequently, molecular diagnostic methods, especially polymerase chain reaction (PCR), have become the preferred approach due to their superior sensitivity, specificity, and ability to detect multiple *T. orientalis* genotypes [15, 16]. Furthermore, DNA sequencing provides valuable information on genetic diversity, evolutionary relationships, and molecular epidemiology, enabling accurate characterization of circulating isolates and comparison with global reference strains [17, 18].

In Iraq, several investigations have documented the occurrence of different *Theileria* species in cattle and other domestic animals [19–22]. However, molecular information regarding *Theileria* infections in ticks remains extremely limited, with only a few published studies investigating tick-associated infections [23]. Moreover, molecular confirmation of *T. orientalis* in Iraqi cattle has been reported only once, from Fallujah City in Al-Anbar Province [24]. To date, no study has simultaneously investigated genetically related *T. orientalis* isolates in cattle and their associated ticks or examined their phylogenetic relationships with globally circulating strains. Consequently, important knowledge gaps remain regarding the molecular epidemiology, host-vector relationship, genetic diversity, and potential transmission dynamics of *T. orientalis* in Iraq.

Despite the increasing recognition of *T. orientalis* as an emerging tick-borne pathogen worldwide, comprehensive molecular epidemiological studies in Iraq remain scarce. Previous investigations have largely focused on detecting *Theileria* species in cattle, while molecular characterization of parasites circulating in naturally infesting ticks has received little attention. Furthermore, no previous Iraqi study has simultaneously evaluated cattle and corresponding tick isolates using sequence-based phylogenetic analysis to determine their genetic relatedness and relationship with international reference isolates. This lack of integrated host-vector molecular data limits the current understanding of transmission pathways, genetic diversity, and regional dissemination of *T. orientalis*, thereby hindering the development of evidence-based surveillance and control strategies.

Therefore, this study aimed to determine the molecular prevalence of *T. orientalis* in naturally infected cattle and their associated ticks from Wasit Province, Iraq, using PCR targeting the *18S rRNA* gene. In addition, the study sought to characterize the genetic diversity of the detected isolates through DNA sequencing, evaluate their phylogenetic relationships with globally reported *T. orientalis* strains, and investigate host-associated risk factors influencing infection. Collectively, these findings provide the first integrated molecular and phylogenetic evidence linking cattle and tick isolates of *T. orientalis* in Wasit Province and establish an important baseline for future epidemiological surveillance and control of oriental theileriosis in Iraq.

## MATERIALS AND METHODS

### Ethical approval

The study protocol was reviewed and approved by the Scientific Committee, College of Veterinary Medicine, University of Wasit, Iraq, and the Institutional Animal Care and Use Committee (IACUC), University of Wasit (Approval No. WU.CVM.CVM-PD-1429; approved on June 25, 2025). All procedures involving animals were conducted in accordance with the institutional guidelines for the ethical care and use of animals in research and complied with internationally accepted principles for veterinary research involving animals. Blood and tick sampling were performed by licensed veterinarians using standard aseptic techniques and appropriate animal restraint to minimize pain, distress, and handling stress. Approximately 5 mL of jugular venous blood was collected using sterile, single-use needles into EDTA-anticoagulated tubes, while attached ticks were carefully removed using sterile forceps to avoid unnecessary tissue injury. All sampled animals were returned to their owners immediately after sample collection without any invasive intervention beyond routine veterinary procedures. Verbal informed consent was obtained from the owners of all participating cattle before sample collection. The study did not involve endangered or protected animal species, and no experimental infections, euthanasia, or procedures that caused prolonged discomfort were performed. Laboratory procedures involving potentially infectious materials were conducted in accordance with appropriate biosafety practices to protect personnel and prevent environmental contamination.

### Study period and location

**Figure 1:**
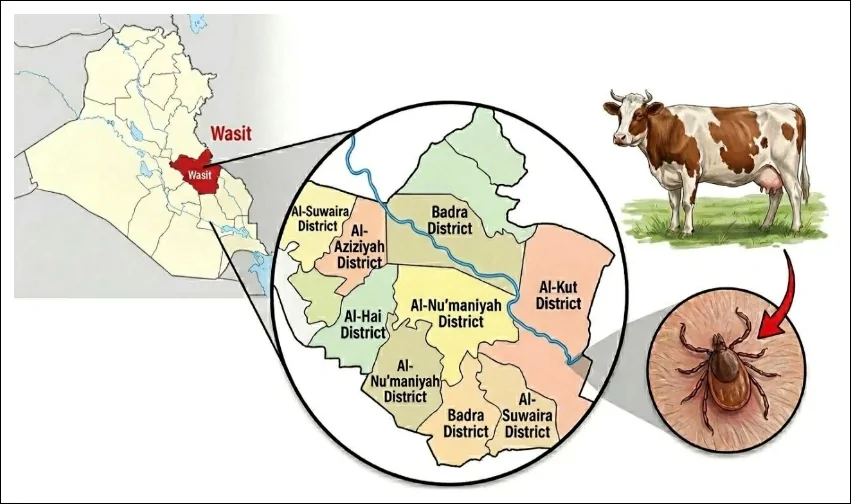
Geographical map of the study areas located in Wasit Province, Iraq.

This cross-sectional study was conducted from July to August 2025 in Wasit Province, located in east-central Iraq, approximately 172 km southeast of Baghdad (Figure 1). The province extends between longitudes 44°40′ E and latitudes 32°00′ N and 33°50′ N and shares its eastern border with the Islamic Republic of Iran. The northeastern region is characterized by low-relief plains and anticlinal folds that receive drainage from adjacent mountainous areas. Wasit Province supports a substantial cattle population owing to favorable agroecological conditions, including access to transboundary marshlands, abundant water resources, and productive alluvial plains that facilitate livestock production [25–27].

### Study design

A cross-sectional molecular epidemiological study was conducted involving 170 naturally tick-infested crossbred cattle selected from different localities of Wasit Province, Iraq. Animals were enrolled following clinical examination and confirmation of natural tick infestation. All cattle were managed under traditional grazing systems on natural pastures and were frequently exposed to ticks despite routine tick-control practices.

The required sample size was calculated according to the formula described by Thrusfield [28]:

*n* = *Z*^2^*P*_exp_ (1-*P*_exp_) / *d*^2^

where:

1. *n* = required sample size;

2. *Z* = 1.96 (95% confidence interval [CI]);

3. *P*<sub>exp</sub> = expected prevalence (0.50);

4. *d* = desired precision (0.075).

### Sample collection

Under aseptic conditions, approximately 5 mL of jugular venous blood was collected from each animal into ethylenediaminetetraacetic acid (EDTA)-anticoagulated tubes. Hard ticks attached to different body regions of each animal were carefully removed using sterile forceps and placed into individually labeled sterile plastic containers. Blood and tick samples were transported to the laboratory under refrigerated conditions and stored at −20°C until molecular analysis. Information on the age and sex of each animal was recorded for subsequent risk-factor analysis.

### Molecular detection of *T. orientalis*

Genomic DNA was extracted from both blood and tick samples using the gSYNC™ DNA Extraction Kit (Geneaid Biotech, New Taipei City, Taiwan) according to the manufacturer's instructions. DNA concentration and purity were evaluated using a NanoDrop spectrophotometer (Thermo Fisher Scientific, Waltham, MA, USA).

Conventional PCR targeting the *18S rRNA* gene was performed using a primer pair designed from the *T. orientalis* Dunya-30-IRAQ reference sequence (GenBank accession no. OQ779975.1) with Primer3Plus (Wageningen University, Wageningen, the Netherlands). The PCR mixture (20 μL) consisted of 5 μL DNA template, 1 μL forward primer, 1 μL reverse primer, and 13 μL nuclease-free water in AccuPower® PCR PreMix tubes (Bioneer, Daejeon, South Korea).

PCR amplification was carried out using a T100 Thermal Cycler (Bio-Rad Laboratories, Hercules, CA, USA) under the following cycling conditions: initial denaturation at 94°C for 7 min; 30 cycles of denaturation at 94°C for 1 min, annealing at 58°C for 30 s, and extension at 72°C for 1 min; followed by a final extension at 72°C for 7 min.

Amplified PCR products were separated by electrophoresis on 1.5% agarose gels stained with ethidium bromide and electrophoresed at 100 V and 80 mA for 90 min. Positive controls consisted of DNA obtained from recently confirmed Iraqi *T. orientalis* isolates [24], whereas nuclease-free water served as the negative control. Amplified products were visualized under an ultraviolet transilluminator, and the expected amplicon size was 505 bp.

### DNA sequencing and phylogenetic analysis

PCR-positive DNA samples from both cattle and tick specimens, together with the corresponding primers, were submitted to Macrogen Inc. (Seoul, South Korea) for bidirectional DNA sequencing using the modified Sanger sequencing method. The obtained nucleotide sequences were edited and aligned using the ClustalW algorithm. Subsequently, representative sequences were submitted to the National Center for Biotechnology Information (NCBI) GenBank database. Phylogenetic relationships and sequence homology analyses were performed using Molecular Evolutionary Genetics Analysis version 11 (MEGA 11).

### Statistical analysis

Statistical analyses were performed using GraphPad Prism version 8 (GraphPad Software Inc., San Diego, CA, USA). The Chi-square (*χ²*) test was used to evaluate differences among categorical variables. Statistical significance was defined as p < 0.05, with significance levels indicated as follows: p* < *0.05 (*), p < 0.01 (**), p < 0.001 (***), and p < 0.0001 (****).

The odds ratio (OR), relative risk (RR), number needed to treat (NNT), and 95% CI were calculated using MedCalc Statistical Software version 22 (MedCalc Software Ltd., Ostend, Belgium) [29].

## RESULTS

### Incidence of tick infestation according to age and sex

Based on clinical examination, the incidence and risk of tick infestation varied significantly according to the age and sex of the study cattle (Table 1). The incidence of tick infestation differed significantly among age groups (p = 0.0149). Cattle aged >3–6 years exhibited the highest infestation rate (36.47%) and RR (0.9388; p = 0.0008), whereas cattle aged <1 year showed the lowest infestation rate (13.53%) and RR (0.2918). Intermediate infestation rates were observed in cattle aged 1–3 years (28.82%) and >6 years (21.18%).

Sex was also significantly associated with tick infestation. Female cattle exhibited a significantly higher infestation rate than males (84.71% vs. 15.29%; p = 0.0039). Likewise, the RR of tick infestation was markedly higher in females (6.3855; p = 0.0001) than in males (0.3335).

**Table 1:** Incidence and risk of tick infestation according to the age and sex of the study cattle (n = 170).

**Factor**	**No. (%)**	**RR**	**NNT**	**95% CI**
Age (years)				
<1	23 (13.53%)	0.2918	3.045 (Benefit)	2.425 (Benefit) to ∞ to 4.089 (Benefit)
1–3	49 (28.82%)	0.6932	7.839 (Benefit)	4.585 (Benefit) to ∞ to 27.004 (Benefit)
>3–6	62 (36.47%)*	0.9388***	42.046 (Benefit)	14.537 (Harm) to ∞ to 8.594 (Benefit)
>6	36 (21.18%)	0.4804	4.366 (Benefit)	3.158 (Benefit) to ∞ to 7.073 (Benefit)
p-value	0.0149	0.0008	–	–
Sex				
Female	144 (84.71%)**	6.3855****	1.4 (Harm)	1.556 (Harm) to ∞ to 1.272 (Harm)
Male	26 (15.29%)	0.3335	3.272 (Benefit)	2.561 (Benefit) to ∞ to 4.528 (Benefit)
p-value	0.0039	0.0001	–	–

RR = Relative risk; NNT = Number needed to treat; CI = Confidence interval.

### Molecular detection of *T. orientalis*

**Figure 2:**
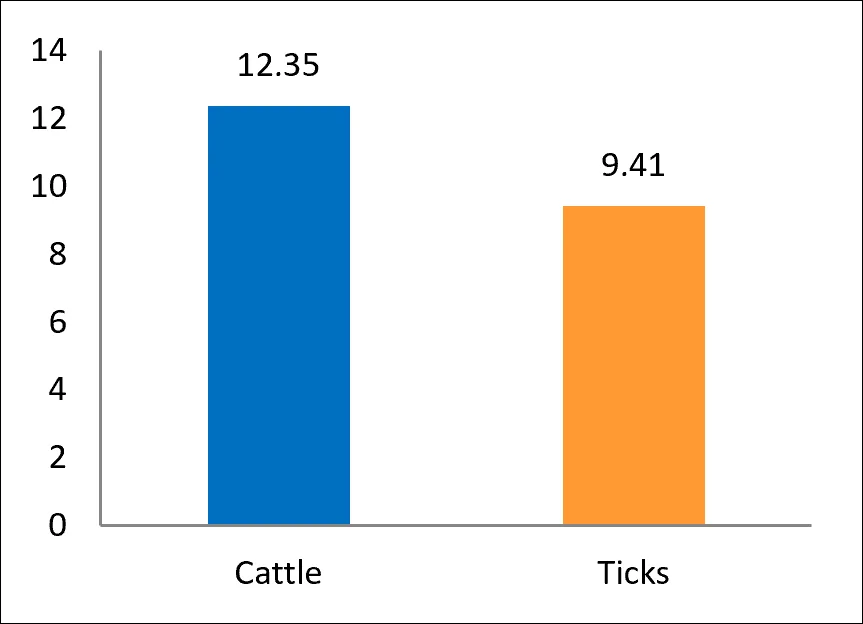
Molecular detection of *Theileria orientalis* infection among cattle and their naturally infesting ticks by polymerase chain reaction.

Conventional PCR detected *T. orientalis* DNA in 21 of 170 (12.35%) cattle blood samples and 16 of 170 (9.41%) corresponding tick samples (Figure 2).

### Distribution of *T. orientalis* infection according to host-related risk factors

The prevalence of *T. orientalis* infection differed significantly among age groups (p = 0.043) (Table 2). The highest prevalence was observed in cattle aged 1–3 years (14.29%) and >3–6 years (14.52%), whereas lower prevalence was observed in cattle aged <1 year (8.70%) and >6 years (8.33%).

Similarly, cattle aged >3–6 years exhibited the highest OR (1.5376) and RR (1.2676), whereas animals aged >6 years showed the lowest OR (0.5859) and RR (0.6496). These findings indicate that cattle aged >3 to 6 years had the greatest likelihood of *T. orientalis* infection.

According to sex, male cattle exhibited a significantly higher prevalence of *T. orientalis* infection than females (19.23% vs. 11.11%; p = 0.0445). Likewise, the OR (1.9048) and RR (1.6129) were significantly greater in males than females (p = 0.0001).

### Genetic characterization and phylogenetic analysis of *T. orientalis*

Sequencing analysis of the detected *T. orientalis* isolates resulted in the submission of nucleotide sequences to the NCBI GenBank database under the isolate designations IS.HA.C1–IS.HA.C21 for cattle isolates and IS.HA.T1–IS.HA.T16 for tick isolates. The corresponding accession numbers ranged from PX851761.1 to PX851781.1 for cattle isolates and from PX851782.1 to PX851797.1 for tick isolates.

**Figure 3:**
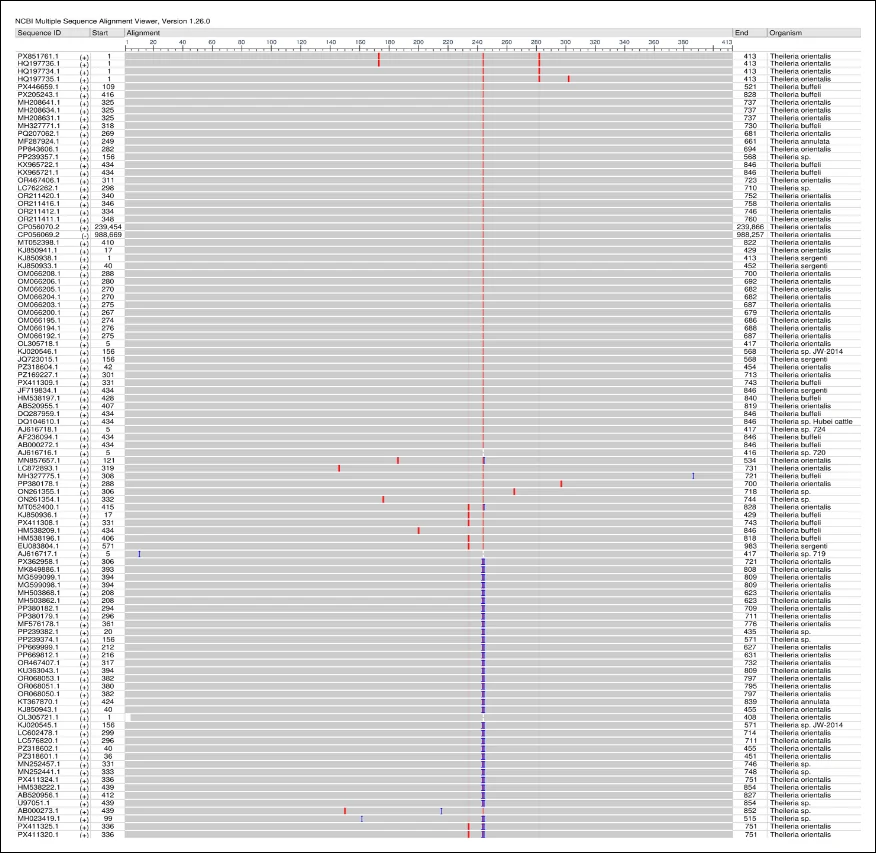
Multiple sequence alignment showing frequency-based nucleotide differences between cattle- and tick-derived *Theileria orientalis* isolates and NCBI-BLAST reference isolates using the NCBI MSA Viewer.

**Figure 4:**
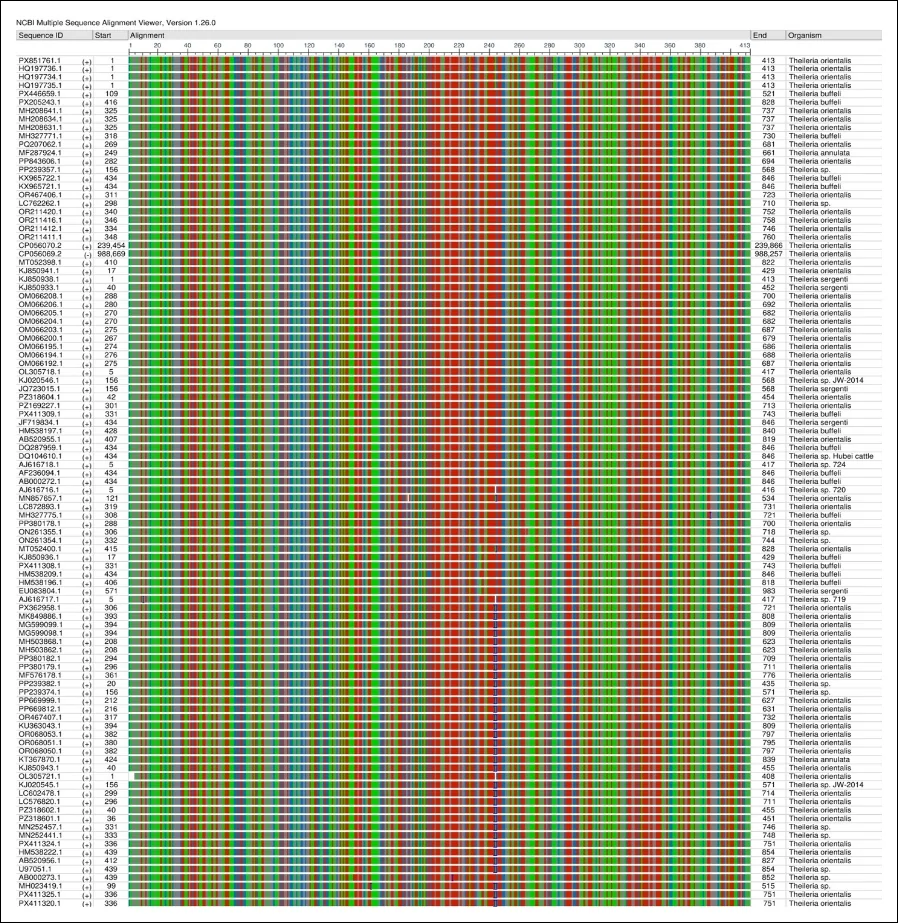
Multiple sequence alignment showing nucleotide sequence alignment of cattle- and tick-derived *Theileria orientalis* isolates and NCBI-BLAST reference isolates using the NCBI MSA Viewer.

**Figure 5:**
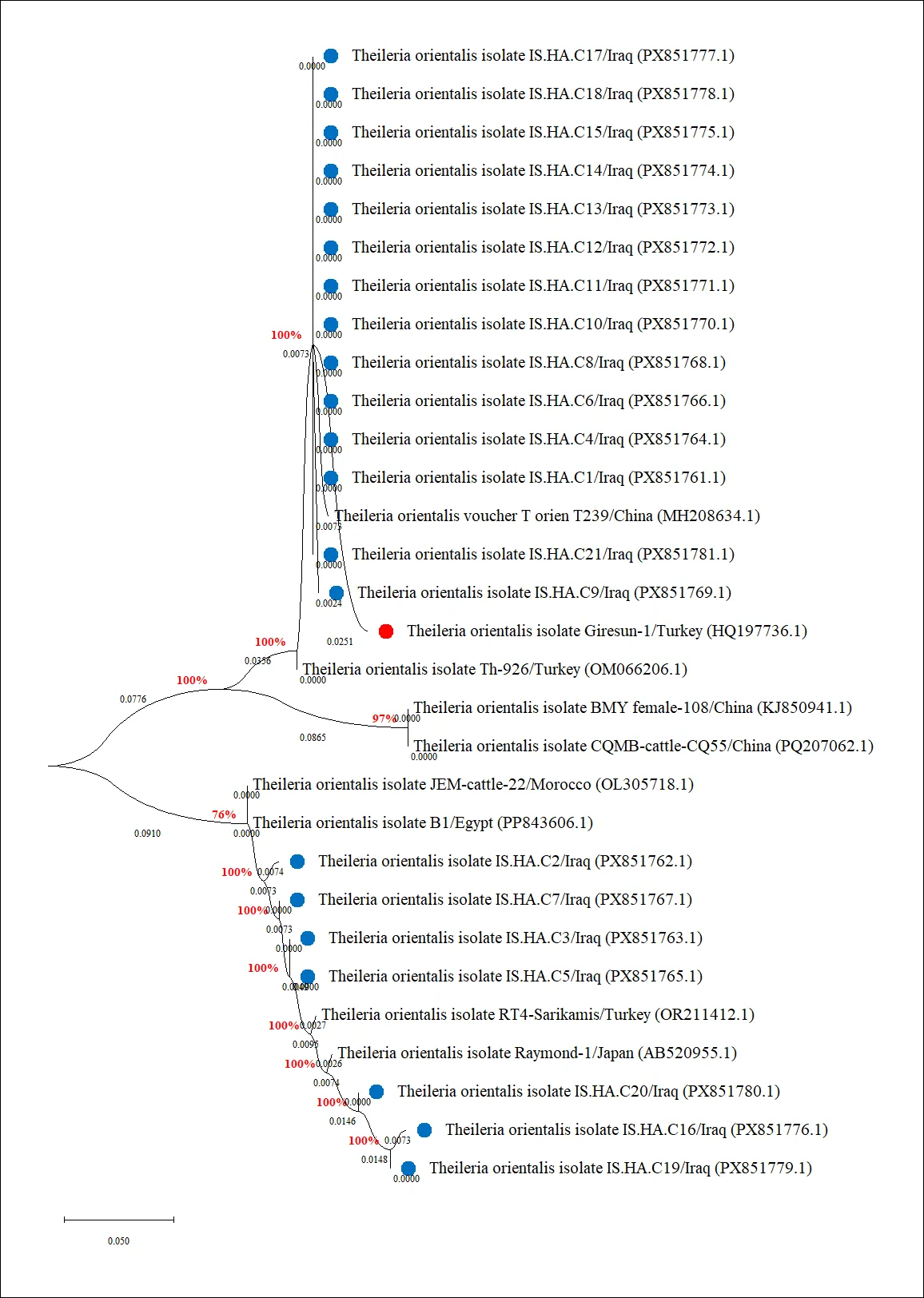
Phylogenetic tree of cattle-derived *Theileria orientalis* isolates and NCBI-BLAST reference isolates. Study isolates are indicated by blue circles, whereas the closely related Turkish isolate (HQ197736.1) is indicated by a red circle.

**Figure 6:**
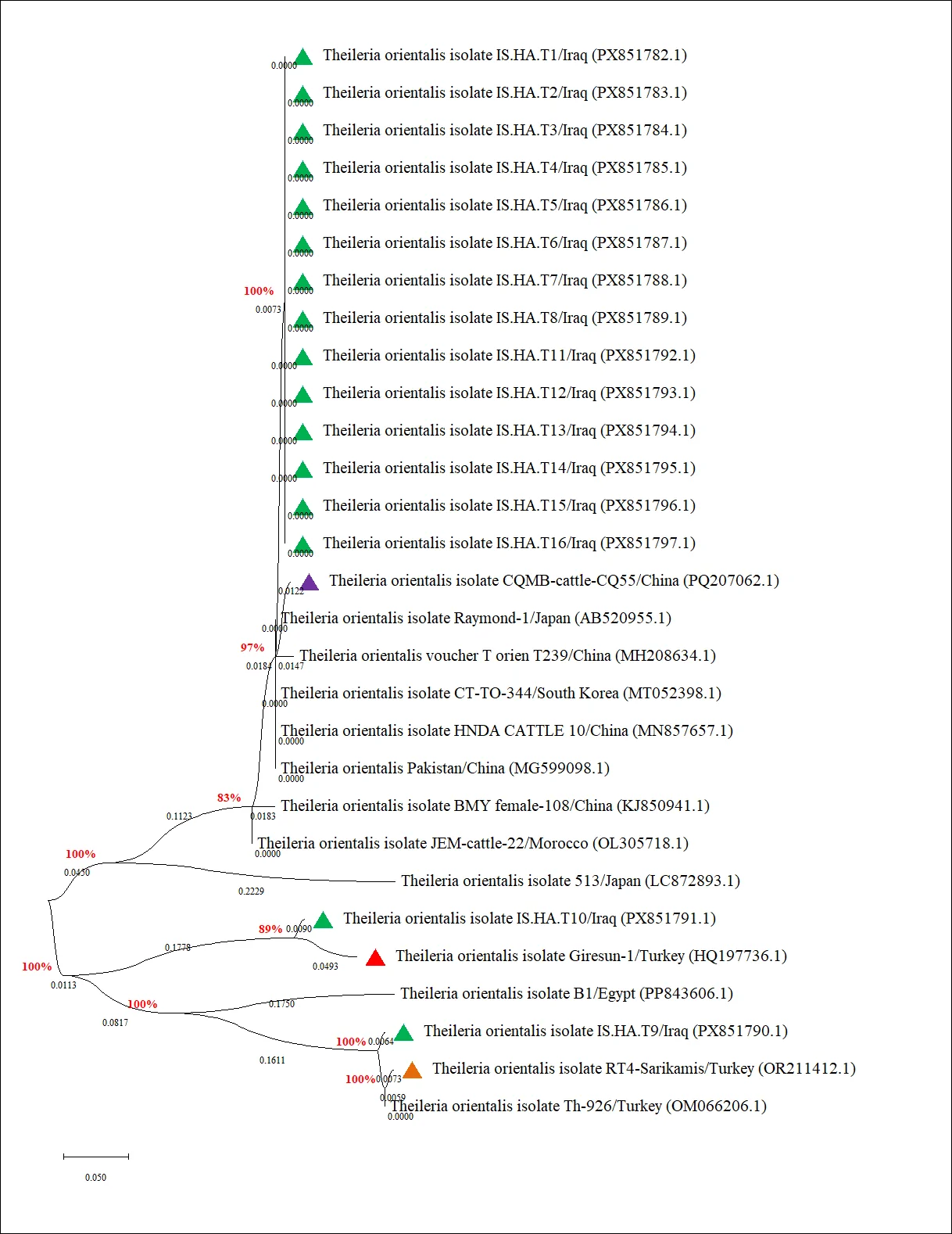
Phylogenetic tree of tick-derived *Theileria orientalis* isolates and NCBI-BLAST reference isolates. Study isolates are indicated by blue circles, whereas the closely related Turkish (red and brown triangles) and Chinese (purple triangle) reference isolates are highlighted.

Comparative sequence analysis showed nucleotide identities of 78.76% to 100% for cattle isolates and 98.16% to 100% for tick isolates, with sequence variation of 0.001% to 0.05%. Phylogenetic analyses performed separately for cattle and tick isolates revealed that all cattle-derived *T. orientalis* isolates clustered closely with the Turkish reference isolate HQ197736.1. In contrast, tick-derived isolates exhibited the highest sequence identity with one Chinese isolate (PQ207062.1) and two Turkish isolates (OR211412.1 and HQ197736.1), suggesting close genetic relationships between Iraqi isolates and geographically neighboring *T. orientalis* strains (Tables 3 and 4; Figures 3–6).

**Table 2:** Distribution of Theileria orientalis infection according to host-related risk factors.

**Factor**	**Total no.**	**Positive, no. (%)**	**OR**	**RR**
Age (years)				
<1	23	2 (8.70%)	0.6416	0.6989
1–3	49	7 (14.29%)*	1.2738	1.2054
>3–6	62	9 (14.52%)*	1.5376*	1.2676*
>6	36	3 (8.33%)	0.5859	0.6496
p-value	–	0.043	0.0001	0.0001
95% CI	–	6.042–16.88	0.2614–17.58	0.4363–14.74
Sex				
Female	144	16 (11.11%)	0.5250	0.6200
Male	26	5 (19.23%)*	1.9048****	1.6129****
p-value	–	0.0445	0.0001	0.0001
95% CI	–	36.42–66.76	7.551–99.81	5.192–74.24

OR = Odds ratio; RR = Relative risk; CI = Confidence interval.

**Table 3:** Homology sequence identity (%) between cattle-derived Theileria orientalis isolates and global NCBI-BLAST reference isolates.

**Local study isolate**				**Global NCBI-BLAST isolate**			
**Name**	**Accession no.**	**Isolate**		**Accession no.**	**Host**	**Country**	**Identity (%)**
IS.HA.C1	PX851761.1	Giresun-1		HQ197736.1	Bovine	Turkey	99.75
IS.HA.C2	PX851762.1	Giresun-1		HQ197736.1	Bovine	Turkey	99.69
IS.HA.C3	PX851763.1	Giresun-1		HQ197736.1	Bovine	Turkey	99.73
IS.HA.C4	PX851764.1	Giresun-1		HQ197736.1	Bovine	Turkey	99.81
IS.HA.C5	PX851765.1	Giresun-1		HQ197736.1	Bovine	Turkey	99.24
IS.HA.C6	PX851766.1	Giresun-1		HQ197736.1	Bovine	Turkey	99.59
IS.HA.C7	PX851767.1	Giresun-1		HQ197736.1	Bovine	Turkey	99.62
IS.HA.C8	PX851768.1	Giresun-1		HQ197736.1	Bovine	Turkey	99.71
IS.HA.C9	PX851769.1	Giresun-1		HQ197736.1	Bovine	Turkey	98.76
IS.HA.C10	PX851770.1	Giresun-1		HQ197736.1	Bovine	Turkey	99.25
IS.HA.C11	PX851771.1	Giresun-1		HQ197736.1	Bovine	Turkey	99.18
IS.HA.C12	PX851772.1	Giresun-1		HQ197736.1	Bovine	Turkey	98.99
IS.HA.C13	PX851773.1	Giresun-1		HQ197736.1	Bovine	Turkey	99.45
IS.HA.C14	PX851774.1	Giresun-1		HQ197736.1	Bovine	Turkey	99.75
IS.HA.C15	PX851775.1	Giresun-1		HQ197736.1	Bovine	Turkey	98.92
IS.HA.C16	PX851776.1	Giresun-1		HQ197736.1	Bovine	Turkey	99.15
IS.HA.C17	PX851777.1	Giresun-1		HQ197736.1	Bovine	Turkey	99.68
IS.HA.C18	PX851778.1	Giresun-1		HQ197736.1	Bovine	Turkey	99.75
IS.HA.C19	PX851779.1	Giresun-1		HQ197736.1	Bovine	Turkey	99.41
IS.HA.C20	PX851780.1	Giresun-1		HQ197736.1	Bovine	Turkey	99.62
IS.HA.C21	PX851781.1	Giresun-1		HQ197736.1	Bovine	Turkey	99.70

**Table 4:** Homology sequence identity (%) between tick-derived T. orientalis isolates and global NCBI-BLAST reference isolates.

**Local study isolate**				**Global NCBI-BLAST isolate**			
**Name**	**Accession no.**	**Isolate**		**Accession no.**	**Host**	**Country**	**Identity (%)**
IS.HA.T1	PX851782.1	CQMB-cattle-CQ55		PQ207062.1	Tick	China	99.83
IS.HA.T2	PX851783.1	CQMB-cattle-CQ55		PQ207062.1	Tick	China	99.83
IS.HA.T3	PX851784.1	CQMB-cattle-CQ55		PQ207062.1	Tick	China	99.83
IS.HA.T4	PX851785.1	CQMB-cattle-CQ55		PQ207062.1	Tick	China	99.83
IS.HA.T5	PX851786.1	CQMB-cattle-CQ55		PQ207062.1	Tick	China	99.83
IS.HA.T6	PX851787.1	CQMB-cattle-CQ55		PQ207062.1	Tick	China	99.83
IS.HA.T7	PX851788.1	CQMB-cattle-CQ55		PQ207062.1	Tick	China	99.83
IS.HA.T8	PX851789.1	CQMB-cattle-CQ55		PQ207062.1	Tick	China	99.67
IS.HA.T9	PX851790.1	RT4-Sarikamis		OR211412.1	Cattle	Turkey	99.73
IS.HA.T10	PX851791.1	Giresun-1		HQ197736.1	Bovine	Turkey	99.64
IS.HA.T11	PX851792.1	CQMB-cattle-CQ55		PQ207062.1	Tick	China	98.16
IS.HA.T12	PX851793.1	CQMB-cattle-CQ55		PQ207062.1	Tick	China	98.87
IS.HA.T13	PX851794.1	CQMB-cattle-CQ55		PQ207062.1	Tick	China	98.82
IS.HA.T14	PX851795.1	CQMB-cattle-CQ55		PQ207062.1	Tick	China	98.97
IS.HA.T15	PX851796.1	CQMB-cattle-CQ55		PQ207062.1	Tick	China	98.86
IS.HA.T16	PX851797.1	CQMB-cattle-CQ55		PQ207062.1	Tick	China	98.82

## DISCUSSION

### Molecular prevalence of *T. orientalis* in cattle and ticks

Information regarding *T. orientalis* infection in cattle remains limited in Iraq [24], although recent studies conducted in Asia [30, 31], Africa [32, 33], the Americas [34], and Europe [35, 36] have demonstrated that this tick-borne parasite is associated with considerable morbidity, production losses, and, in some cases, mortality in cattle. In the present study, the molecular prevalence of *T. orientalis* was 12.35% in cattle and 9.41% in their naturally infesting ticks. These findings provide the first simultaneous molecular evidence of *T. orientalis* infection in cattle and associated ticks from Wasit Province, Iraq.

Compared with previous reports, the prevalence observed in this study was higher than those reported from Ethiopia (2.2%) [37], Turkey (5.6%) [38], and Egypt (8.8%) [39], but was comparable with reports from Vietnam (13.8%) [40] and Pakistan (15.0%) [41]. Lower prevalence than the present study has also been reported in northeastern Thailand (30.1%) [42], Kazakhstan (33.3%) [43], China (14.27%–36.5%) [30, 44], Malaysia (49.76%) [45], Japan (10%–64.8%) [46], and India (83.3%) [31]. Such wide geographical variation in the prevalence of bovine theileriosis is likely attributable to differences in climatic conditions, tick vector abundance, cattle management systems, host immunity, diagnostic approaches, and the genetic diversity of circulating *Theileria* strains [47].

Environmental factors strongly influence tick ecology, with warm and humid climates generally favoring tick survival and transmission, thereby increasing disease prevalence [48]. In addition, farm management practices, including grazing systems, biosecurity measures, and nutritional status, may alter host susceptibility by increasing physiological stress [49]. Farms managed as small-scale or sideline enterprises often have limited resources for tick control, hygiene, and routine veterinary surveillance, which may further increase the risk of blood parasite transmission [50]. Moreover, genetic variation among *Theileria* species, including the emergence of strains with different levels of virulence or antimicrobial resistance, may further contribute to regional differences in disease epidemiology [51]. Variability in reported prevalence may also reflect differences in molecular diagnostic procedures, including pre-analytical sample handling, analytical protocols, and biological factors affecting parasite detection.

### Association of *T. orientalis* infection with host-related risk factors

The present study demonstrated that *T. orientalis* infection was significantly associated with animal age. The highest prevalence was observed in cattle aged 1–3 years and >3–6 years, whereas the highest risk of infection was detected in cattle aged >3–6 years. Likewise, the prevalence of PCR-positive tick samples was greatest among cattle aged 1–3 years. Interpretation of age-related susceptibility should be approached with caution because previous studies have not consistently distinguished between epidemic and endemic disease conditions [6, 52–54].

Previous investigations have reported inconsistent findings regarding the influence of age on *T. orientalis* infection. In Australia, *T. orientalis* was detected microscopically in a 4-day-old calf and by quantitative PCR in both newborn and fetal calves [55, 56]. Similarly, Mekata *et al.* [57] reported that calves born to infected dams were PCR-negative during the first 30 days after birth, whereas 88% became infected by 4 months of age, suggesting that vertical transmission may require several months before becoming detectable under low tick challenge conditions.

In Malaysia, Ola-Fadunsin *et al.* [45] reported no significant difference in infection prevalence between cattle aged ≤1 year (44.5%) and 1–≤2 years (42.22%), whereas significantly higher prevalence was observed in animals aged 2–≤5 years (52.52%) and >5 years (59.64%). Likewise, Selim *et al.* [39] demonstrated that asymptomatic cattle older than 3 years had a significantly greater prevalence of *T. orientalis* infection (13.1%; p < 0.003) than cattle aged <1 year (1.6%) or 1–3 years (5.6%). Conversely, cattle residing in endemic regions, including Iraq, may gradually develop partial protective immunity following repeated exposure to infected ticks, resulting in persistent infection without overt clinical disease [14, 20, 58, 59]. Therefore, higher prevalence in older animals may reflect cumulative exposure rather than increased susceptibility to clinical theileriosis.

### Effect of sex on *T. orientalis* infection

Male cattle exhibited significantly higher infection prevalence, OR, and RR than female cattle. In contrast, the prevalence of *T. orientalis* infection in ticks was not significantly associated with host sex, although ticks collected from male cattle showed a numerically higher risk of infection.

Previous studies have reported inconsistent associations between sex and *T. orientalis* infection. Selim *et al.* [39] found no significant difference between female (9.2%) and male (7.5%) cattle, whereas Ola-Fadunsin *et al.* [45] reported a higher prevalence among females (51.33%) than males (43.89%). The discrepancy between the present findings and previous reports may reflect differences in herd structure, management practices, and animal utilization. In the present study, most female cattle were maintained for dairy production, whereas male cattle were primarily reared for beef production under more extensive grazing conditions, which may increase their exposure to tick vectors.

### Phylogenetic relationships of Iraqi *T. orientalis* isolates

Although *T. orientalis* has previously been reported in neighboring countries, including Turkey, Iran, and Pakistan, molecular phylogenetic information from Iraq has been lacking despite the country's strategic geographical position linking Asia and the Mediterranean region. In the present study, phylogenetic analysis demonstrated that all cattle-derived *T. orientalis* isolates clustered closely with the Turkish isolate HQ197736.1. In contrast, tick-derived isolates exhibited the greatest sequence identity with one Chinese isolate (PQ207062.1) and two Turkish isolates (OR211412.1 and HQ197736.1).

These findings suggest regional genetic connectivity and may reflect transboundary movement of infected animals or tick vectors. The greater genetic diversity observed among tick isolates may be explained by ticks' ability to acquire parasites from multiple infected hosts throughout their life cycle, allowing them to harbor a broader range of *T. orientalis* genotypes than those circulating within individual cattle populations. Recent outbreaks reported in Asia and Australia have been associated with highly pathogenic genotypes, including the Ikeda genotype, which has expanded into previously unaffected regions [6]. Consequently, the present findings contribute important baseline molecular evidence regarding the circulation and potential dissemination of *T. orientalis* lineages in Iraq. However, the statement regarding "haplotype diversity and network analysis showing shared versus unique haplotypes between cattle and ticks" should be removed because such analyses were not performed in this study.

### Utility of the *18S rRNA* gene and study limitations

The *18S rRNA* gene remains one of the most reliable molecular markers for detecting *T. orientalis* because of its high copy number and the presence of conserved regions interspersed with hypervariable domains that enable accurate differentiation from other apicomplexan parasites [60–62]. Furthermore, the conserved nature of the *18S rRNA* gene facilitates phylogenetic reconstruction and comparison of *Theileria* isolates worldwide, providing a robust framework for investigating evolutionary relationships and genotype diversity [63–66].

Nevertheless, several limitations should be acknowledged. The study was limited to a single province in Iraq, which may not fully represent the nationwide epidemiology of *T. orientalis*. In addition, phylogenetic characterization was based solely on the *18S rRNA* gene. More discriminatory genetic markers, such as the *MPSP* gene, *ITS* regions, mitochondrial genes, or whole-genome sequencing, were not investigated. Consequently, genotype-specific characterization, identification of polymorphic sites, and differentiation of major genotypes, including Ikeda, Chitose, and Buffeli, were beyond the scope of the present study. Future nationwide molecular surveillance incorporating multiple genetic markers and larger sample sizes is therefore warranted to improve understanding of the genetic diversity, transmission dynamics, and epidemiology of *T. orientalis* in Iraq.

## CONCLUSION

This study provides the first comprehensive molecular evidence of *T. orientalis* infection in cattle and their naturally infesting ticks from Wasit Province, Iraq, and the first phylogenetic characterization linking host- and vector-derived isolates in the country. Conventional PCR detected *T. orientalis* in 12.35% of cattle and 9.41% of tick samples. Infection was significantly associated with host age and sex, with cattle aged >3–6 years exhibiting the highest risk of infection, whereas male cattle showed a significantly higher prevalence and risk of infection than females. Phylogenetic analysis based on the *18S rRNA* gene demonstrated a high degree of genetic conservation among Iraqi isolates, with cattle isolates clustering closely with a Turkish reference isolate and tick isolates showing close genetic relationships with both Turkish and Chinese isolates, indicating possible regional circulation of genetically related *T. orientalis* lineages.

From a practical perspective, these findings provide valuable baseline molecular epidemiological data that can support the development of evidence-based surveillance programs, risk-based tick-control strategies, and early molecular diagnosis of oriental theileriosis in Iraq. Simultaneous molecular investigation of cattle and their associated ticks also improves understanding of host-vector relationships and may facilitate more effective integrated control programs to reduce production losses from tick-borne diseases.

A major strength of this study is the combined evaluation of host- and vector-derived samples using molecular detection, DNA sequencing, and phylogenetic analysis, thereby generating the first integrated molecular dataset for *T. orientalis* in Iraq.

Future studies should include larger nationwide surveys involving different ecological regions, cattle breeds, and tick species while incorporating more discriminatory molecular markers, such as *MPSP*, *ITS*, mitochondrial genes, or whole-genome sequencing, to characterize circulating genotypes and clarify transmission dynamics. Longitudinal investigations integrating vector ecology, genotype distribution, and clinical outcomes will further enhance understanding of the epidemiology of *T. orientalis* and support sustainable control strategies for the Iraqi cattle industry. Overall, this study establishes an important molecular foundation for future surveillance and contributes to the growing body of knowledge on the epidemiology and phylogenetic diversity of *T. orientalis* in the Middle East.

## DATA AVAILABILITY

All data are included within the manuscript.

## GENERATIVE AI DECLARATION

The authors declare that no generative artificial intelligence or AI-assisted technologies were used in the writing, analysis, or preparation of this manuscript

## AUTHORS’ CONTRIBUTIONS

AHKS: Conceived and designed the study, coordinated field investigations, collected tick samples, and participated in data acquisition. GJKA: Collected blood samples and contributed to field sampling and specimen handling. TKA: Performed molecular laboratory analyses of blood samples. IME: Conducted molecular laboratory analyses of tick samples. ZA: Prepared the initial manuscript draft and contributed to data interpretation. HAJG: Performed statistical analyses, conducted sequence submission to GenBank, carried out phylogenetic and bioinformatics analyses of cattle and tick isolates, supervised the study, and critically revised the manuscript. All authors read, reviewed, and approved the final version of the manuscript.
